# A dune with a view: the eyes of a neotropical fossorial lizard

**DOI:** 10.1186/s12983-019-0320-2

**Published:** 2019-06-10

**Authors:** Carola A. M. Yovanovich, Michele E. R. Pierotti, Miguel Trefaut Rodrigues, Taran Grant

**Affiliations:** 1 Department of Zoology, Institute of Biosciences, University of São Paulo, São Paulo, Brazil; 2 Naos Marine Laboratories, Smithsonian Tropical Research Institute, Panama City, Panama

**Keywords:** Fossoriality, Gymnophthalmidae, Oil droplets, Photoreceptors, Retina, São Francisco River, Teiidae, Transmittance, Visual ecology, Visual system

## Abstract

**Background:**

Lizards are excellent models to study the adaptations of the visual system to different scenarios, and surface-dwelling representatives have been relatively well studied. In contrast, very little is known about the functional anatomy of the eyes of fossorial lineages, and properties such as the light transmission by the ocular media have never been characterised in any fossorial species. Some lizards in the family Gymnophthalmidae endemic to the sand dunes of North Eastern Brazil have evolved sand-burrowing habits and nocturnal activity. Lizards in the sister group to Gymnophthalmidae, the family Teiidae, have decidedly diurnal and epigeal lifestyles, yet they are equally poorly known in terms of visual systems. We focussed on the eye anatomy, photoreceptor morphology and light transmittance properties of the ocular media and oil droplets in the gymnophthalmid *Calyptommatus nicterus* and the teiid *Ameivula ocellifera*.

**Results:**

The general organisation of the eyes of the fossorial nocturnal *C. nicterus* and the epigeal diurnal *A. ocellifera* is remarkably similar. The lenses are highly transmissive to light well into the ultraviolet part of the spectrum. The photoreceptors have the typical cone morphology, with narrow short outer segments and oil droplets. The main difference between the two species is that *C. nicterus* has only colourless oil droplets, whereas *A. ocellifera* has colourless as well as green-yellow and pale-orange droplets.

**Conclusions:**

Our results challenge the assumption that fossorial lizards undergo loss of visual function, a claim that is usually guided by the reduced size and external morphology of their eyes. In the case of *C. nicterus*, the visual system is well suited for vision in bright light and shows specialisations that improve sensitivity in dim light, suggesting that they might perform some visually-guided behaviour above the surface at the beginning or the end of their daily activity period, when light levels are relatively high in their open dunes habitat. This work highlights how studies on the functional anatomy of sensory systems can provide insights into the habits of secretive species.

## Background

The evolutionary history of squamate reptiles is rich in transitions regarding microhabitat use and diel patterns [1, 2], both of which pose particular challenges to the performance of the visual system. This makes them an excellent system to study the range of adaptations selected for different scenarios, and phenotype convergences among species that have evolved towards the same lifestyle.

In the 1930s, the seminal work of Gordon Walls proposed pathways for the evolution and adaptation of the eyes of snakes at the optical and retinal structure levels [3]. More recently, data on spectral sensitivities, opsin gene expression and light transmittance of ocular media have complemented his pioneering research, shedding further light on the ecology of ancestral snakes [4–7]. Among lizards, the taxonomic and ecological coverage of research about the structure and function of the visual system is rather patchy. Gekkotans have been the major focus of investigation due to their nocturnal lifestyle and subsequent transition to diurnality in some groups [3, 8–17]. Walls surveyed a few other nocturnal lizards from different phylogenetic groups, such as the scincoid *Xantusia* and the anguimorph *Heloderma* [3]. There are also several studies dealing with “typical” diurnal, four-limbed lacertiform lizards, including iguanians (e.g., anoles, dragon lizards, chameleons) [18–27]-, skinks [28–31], and lacertids [22, 32, 33].

**Fig. 1:**
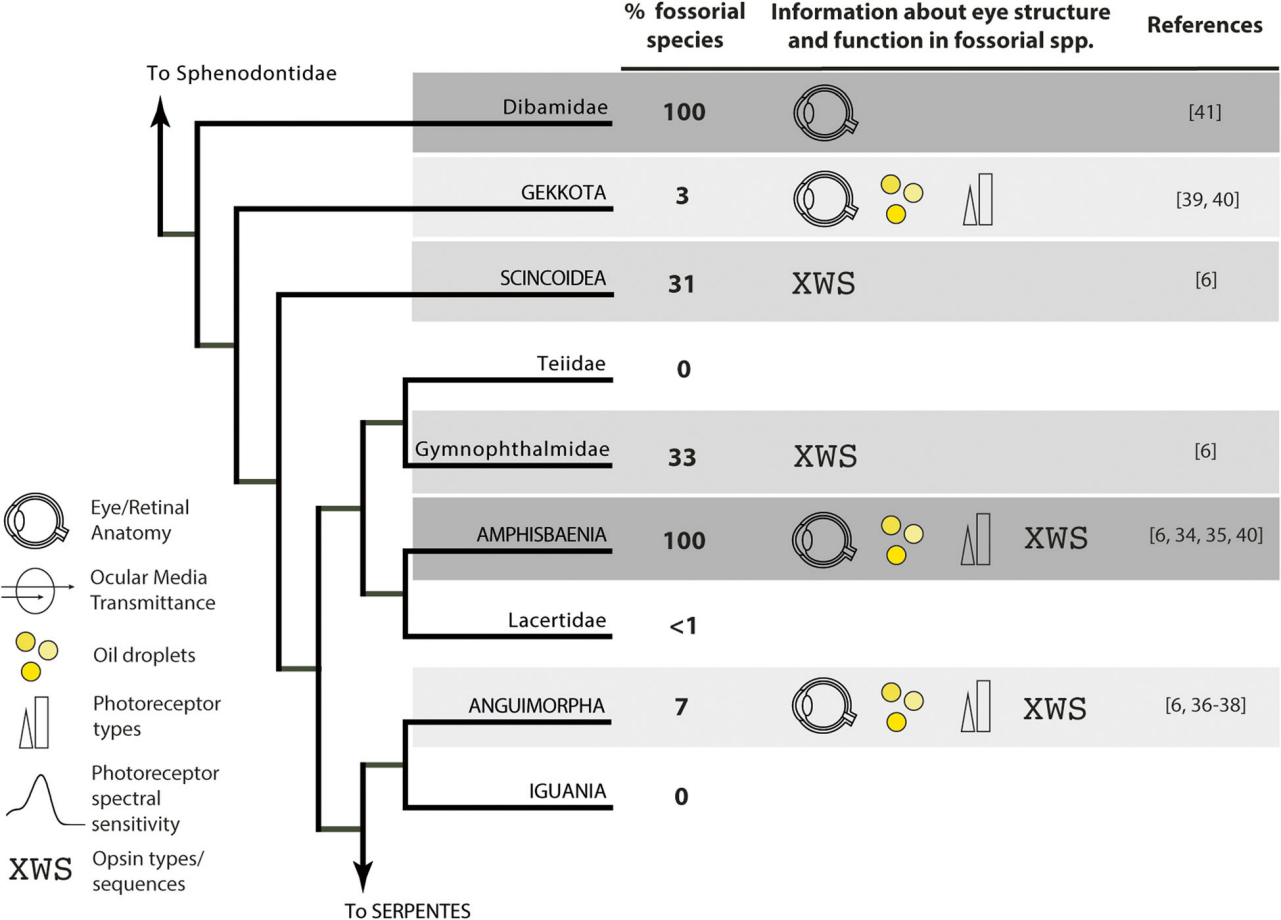
Overview of the current knowledge of the eyes of fossorial lizards in a phylogenetic context. The tree topology and nomenclature are from [42] (several supra-familiar clades were collapsed for simplicity). The percentages of fossorial species were calculated from [2]. Clades with similar proportions of fossorial species are coded in grey scale to ease visualization. To the best of our knowledge, there are no published data on ocular media transmittance and photoreceptor spectral sensitivities for any fossorial lizard. Eyeball cartoon by Laymik from Noun Project

In contrast, even though fossoriality has evolved independently in several lineages of lizards, research on their visual systems is almost entirely morphological (with the exception of a recent dataset about opsin sequences of seven species), as shown in Fig. 1 [6, 34–41]. In addition to the lack of information about spectral sensitivities and light transmittance in the eyes of fossorial lizards in general, there is also a striking gap in knowledge of even basic eye anatomy and retinal histology of Scincoidea and Gymnophthalmidae. The latter clade is particularly relevant because its sister group, Teiidae, has been entirely overlooked in studies of the visual system.

Gymnophthalmids and teiids are primarily diurnal and surface-dwelling [1, 2], but gymnophthalmids have undergone several independent events of transition to a fossorial lifestyle [43]. Among them, the species that inhabit the continental dunes of the middle São Francisco River in the semiarid Brazilian Caatinga are particularly interesting. This area is rich in endemic species of lizards and snakes, many of which present striking adaptations to psammophilous environments [44]. Contrary to other fossorial lizards that burrow in relatively firm soil [45], these animals burrow in the loose sand, so their visual environment can be quite different in terms of openness and light availability, which might be reflected in a particular set of adaptations of the visual system.

The most extreme cases of fossoriality (meant as living primarily, but not exclusively, underground) among gymnophthalmids are found in *Calyptommatus*, one of the genera endemic to the middle São Francisco River. Its four species are similar and share an elongate body, short tail, absence of external forelimbs, and hind limbs reduced to styliform appendages [46], among other traits associated with fossoriality. They are most of the time superficially buried under the sand, both when active and inactive, although they occasionally raise the head above ground for short periods, and can sometimes be found on the surface hiding under leaf litter [46]. Additionally, all of them are active at night [46], which probably evolved to avoid the extreme high temperatures of daytime in their habitats [47].

The gross external morphology of these lizards shows the typical adaptations that protect the ocular region from mechanical abrasion in fossorial species, including non-protrusive, small eyes covered by a spectacle replacing the eyelids [46]. A recent study of the ontogeny and morphology of the eyelids in limbless and typical lacertiform gymnophthalmids revealed a pattern that reflects the transition to fossoriality in the group. *Calyptommatus sinebrachiatus* showed the most extreme case of specialisation, including absence of the nictitating membrane, fusion of eyelids into spectacle, and loss of the anterior chamber between the cornea and the lens [48]. However, the authors did not provide details on the features of the fine retinal structure that influence performance in bright or dim light environments, such as morphological and spectral types of photoreceptors and oil droplets.

**Fig. 2:**
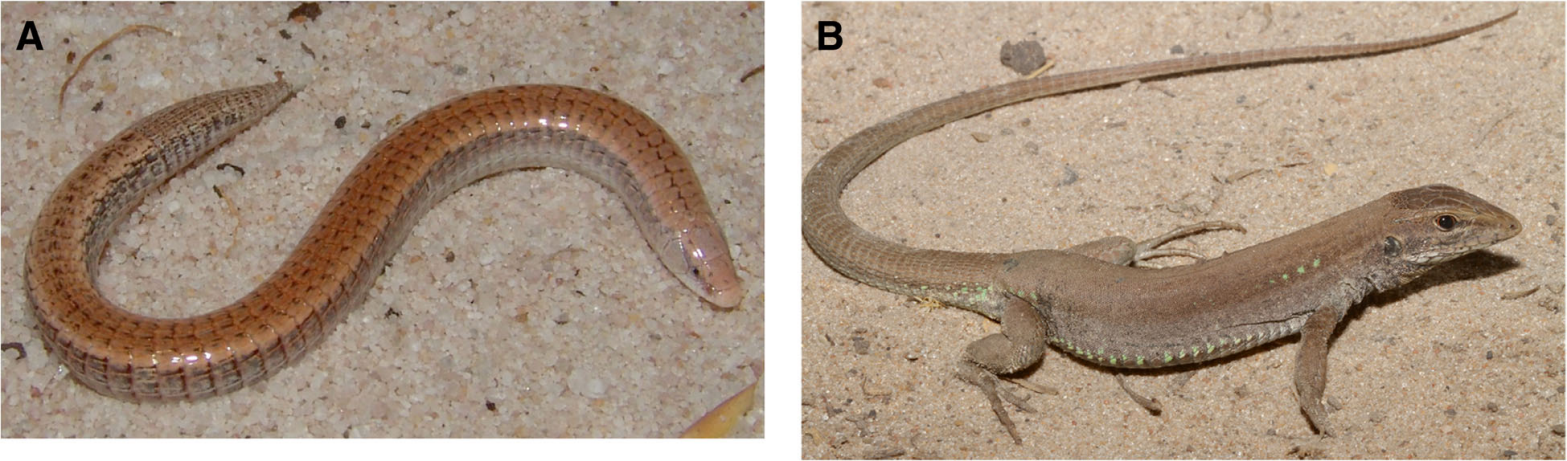
Live specimens from the species used in this study. **a**. *Calyptommatus nicterus*. Note the absence of limbs and the small, non-protruding eyes. **b**. *Ameivula ocellifera*

In this study, we aim to determine the extent to which the eyes of *Calyptommatus nicterus* Rodrigues 1991 (Fig. 2a) reflect its fossorial and nocturnal lifestyle by examining their general anatomy, light transmittance properties of the ocular media, and photoreceptor and oil droplet morphological and spectral types. Given the lack of this kind of information for closely related species with more typical diurnal and surface-dwelling habits, we also include the sympatric species *Ameivula ocellifera* Spix 1825 (Fig. 2b), as a representative of Teiidae.

## Results

### Eye morphology and retinal structure

**Fig. 3:**
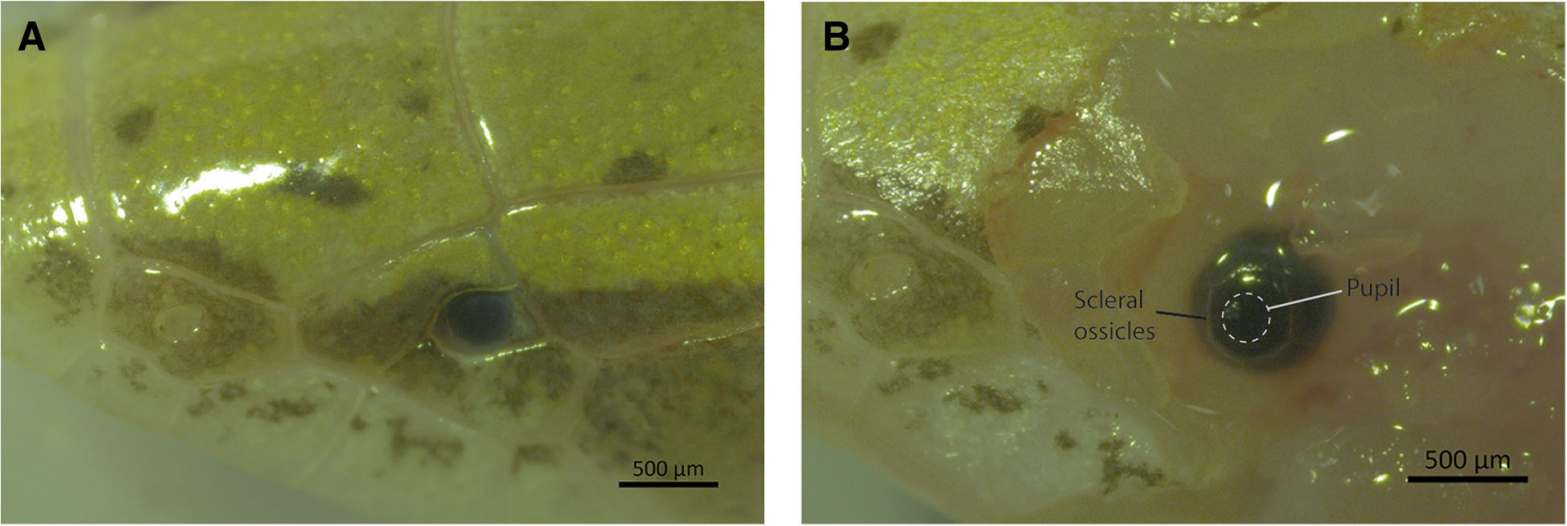
External anatomy of the eye of *Calyptommatus nicterus*. **a**. Recessed position of the eye in the intact head. **b**. Eye exposed during dissection

The eye of *Calyptommatus nicterus* is visible as a small, black dot beneath the transparent spectacle (Fig. 3a). Closer inspection after removal of the spectacle and adjacent tissues reveals a roughly spherical shape with a transverse length of approximately 600 μm. The edges of the scleral ossicles are visible as a clear ring surrounding the cornea. The iris is black and surrounds a round pupil beneath which the lens can be seen (Fig. 3b).

**Fig. 4:**
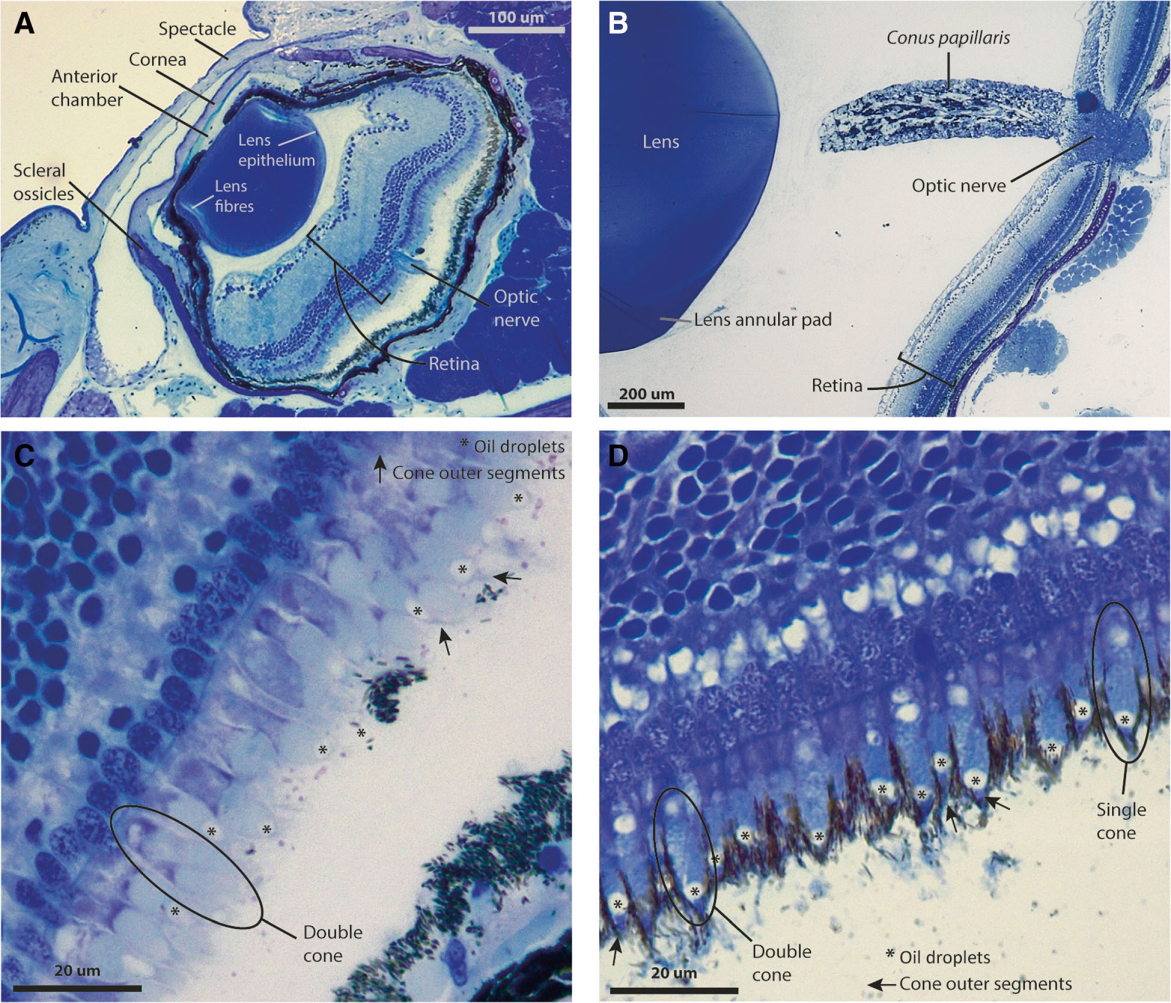
Internal ocular anatomy and retinal structure in *Calyptommatus nicterus* and *Ameivula ocellifera*. **a**, **c**: *C. nicterus*. **b**, **d**: *A. ocellifera*. Note the similarity in retinal thickness and cell sizes between species, despite the difference in eye size

The histological sections show that the lens is separated from the cornea by the anterior chamber and lacks an annular pad. Despite its minuscule size, lying in the range of 100–200 μm in diameter, the lens is well structured with concentric fibres and lens epithelium (Fig. 4a). The retina is thick in relation to the eyecup size, leaving little space to be filled with vitreous. Its overall organisation is typical for a functional vertebrate eye, with pigment epithelium, photoreceptors, two layers of neurons and a well-developed optic nerve (Fig. 4a). The *conus papillaris* is absent and there is no indication of a fovea. In the case of *Ameivula ocellifera*, an annular pad surrounds the lens and a *conus papillaris* protrudes from the head of the optic nerve. The retina is proportionately much thinner than in *C. nicterus*, although similar in absolute thickness and in lacking a fovea (Fig. 4b).

The photoreceptors of *C. nicterus* have typical cone morphology characterised by tapered outer segments, with oil droplets in some single cones and in the principal member of the double cones. The outer segments are narrow, very short, and usually have detached from the inner segment and are retained between the cytoplasmic projections of the pigment epithelial cells (Fig. 4c). The photoreceptor nuclei are arranged in a single layer. The photoreceptor layer of the retina of *Ameivula ocellifera* is also composed of single and double cones with oil droplets and outer segments similar in size and shape to those of *C. nicterus* (Fig. 4d).

### Oil droplets

**Fig. 5:**
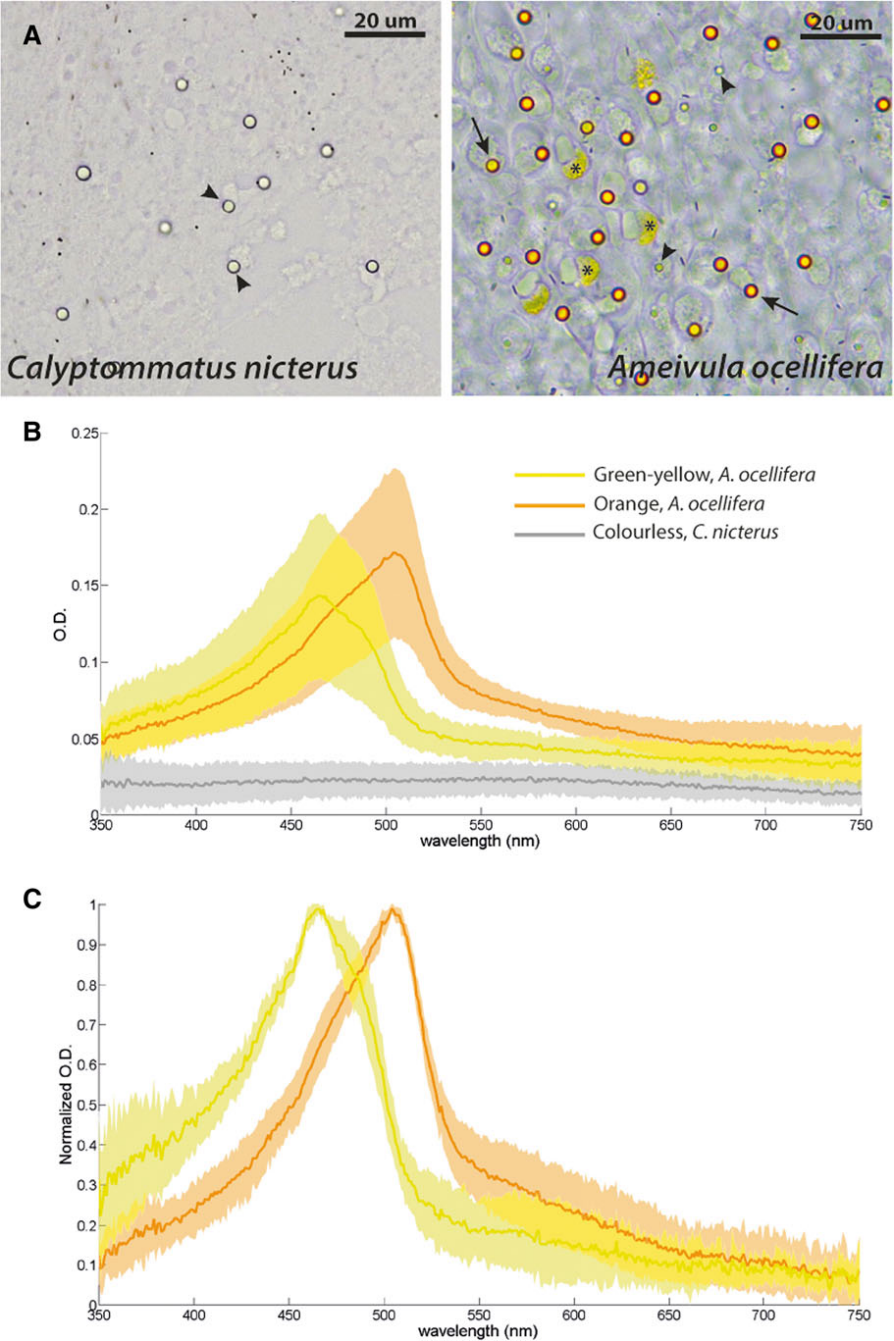
Characterisation of retinal oil droplets in *C. nicterus* and *A. ocellifera*. **a**: Appearance of oil droplets in freshly extracted retinas. Asterisks: Diffuse pigment in cone inner segment; arrowheads: colourless oil droplets; arrows: coloured oil droplets. **b**: Optical density and **c**: normalized optical density from MSP recordings of isolated oil droplets. Average spectra (continuous line) and standard deviations (shaded region) obtained from two eyes from each species are shown for each class of oil droplets

The freshly extracted retinas of *Calyptommatus nicterus* observed under the light microscope show that the oil droplets vary considerably in size without any clear distribution pattern, yet all of them look colourless. Absorbance measurements using microspectrophotometry (MSP) confirmed that they have typically flat spectra and optical densities (OD) below OD = 0.05 (Fig. 5a, b). In *Ameivula ocellifera*, the size range of the oil droplets is similar, but different spectral types are present. The smallest oil droplets look colourless, as in *C. nicterus*, whereas the larger ones look pale green-yellow or pale orange (Fig. 5a). MSP recordings from this species showed that the mean peak optical density for the green-yellow oil droplets is OD = 0.144 (*n* = 8; s.d. 0.054; range 0.086–0.247), whereas for the pale orange ones it is OD = 0.173 (*n* = 15; s.d. 0.055; range 0.085–0.269) (Fig. 5b). The mean cut-off wavelengths calculated from normalized ODs (Fig. 5c) were λc = 483.7 (*n* = 7; mode = 482; range 475–491) for the green-yellow oil droplets and λc = 505.8 (*n* = 15; mode = 507; range 499–511) for the pale orange ones.

### Ocular media transmittance

**Fig. 6:**
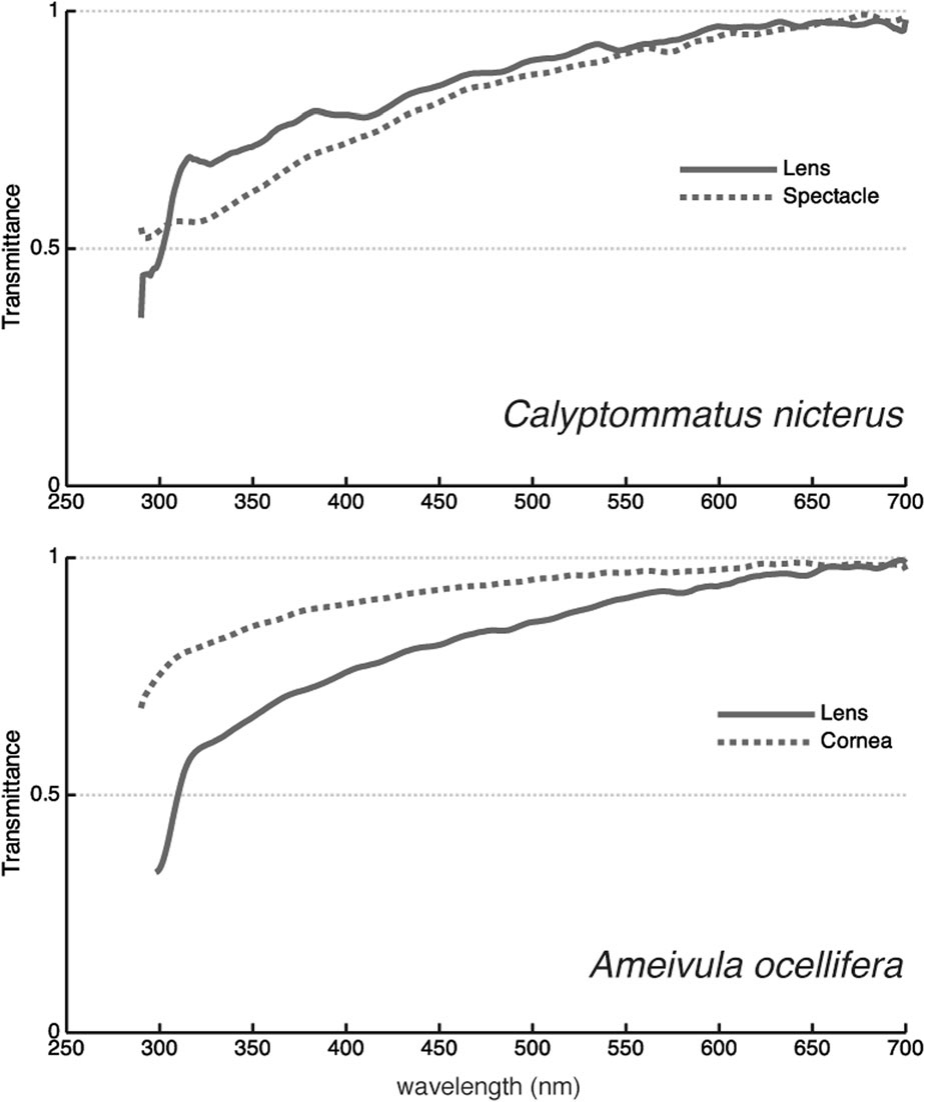
Ocular media transmittance of *Calyptommatus nicterus* (*n* = 2) and *Ameivula ocellifera* (*n* = 2). Note the similarity in the lens transmittance patterns of the two species despite the striking differences in eye size and lifestyle

The lenses of both species are transparent upon direct observation and transmit almost all light in the visible and near ultraviolet (UV) part of the spectrum when measured with a spectrometer (Fig. 6). The wavelength at which the light transmittance is 50% of the maximum (λ_T50_) was 303 nm for *C. nicterus* and 310 nm for *A. ocellifera*. In the latter species, the cornea is even more transmissive (Fig. 6), making this eye highly reachable by UV light. The eyes of *C. nicterus* are very challenging to dissect due to their tiny size, so it was not possible to measure isolated corneas from this species. However, we were able to measure the spectacle, which constitutes an additional layer in the optical pathway, and found that its transmittance pattern is very similar to that of the lens (Fig. 6).

## Discussion

Fossorial lizards have often been assumed to undergo loss of visual function based on their eye external morphology and general internal structure [35, 49], overlooking features of the retinal anatomy that can be more informative about how the eyes can perform in the visual environments set by their microhabitats and diel activity patterns. Here we studied the functional anatomy of the eyes of *Calyptommatus nicterus*, showing that despite being significantly reduced in size, the eyes of fossorial lizards can exhibit the same general organisation as those of surface-dwelling lizards, albeit departing from the most typical diurnal configuration in a number of ways.

The absence of the *conus papillaris* in *C. nicterus* is shared with almost every other fossorial lizard, and most probably is related to the reduction in eye size [36]. The lack of a fovea is expected in *C. nicterus*, as many other nocturnal lizards also lack it [36]. However, as we could not identify a fovea in the eyes of *A. ocellifera* either, the relative contributions of lifestyle and phylogeny to its absence are unclear. Further studies sampling more species in both groups will be needed to clarify this point. The fact that the retinal layers are as well-structured in *C. nicterus* as in *A. ocellifera* and the presence of an optic nerve suggest that the connectivity of the eyes to the visual processing centres in the brain is not impaired in the fossorial species. Further studies involving electrophysiology and retinal topography mapping would provide additional insights into the visual processing abilities of this lizards.

The photoreceptors in the retina of *C. nicterus* look virtually identical to those of *Ameivula ocellifera* (Fig. 4c, d), and both of them match Wall’s “typical cones” of diurnal lizards [3] in that they can be single or double, they possess oil droplets, and their outer segments are very narrow and short and completely embedded in the pigment granules from the retinal pigment epithelium. We found no evidence of rod photoreceptors in either species. However, their presence cannot be ruled out considering that the pigment granules obscure many outer segments preventing their inspection, and that rhodopsin RNA was successfully sequenced in the gymnophthalmid *Bachia* cf. *flavescens* [6]. In situ hibridisation aimed at rhodopsin and different cone opsins mRNA in the inner segments would be a suitable approach to work around the lack of clues from the outer segments and clarify the identity of all photoreceptors in these species.

The morphology of photoreceptors is quite plastic in squamates and is indeed at the core of Walls’ transmutation theory, so it is puzzling that *C. nicterus* does not have enlarged, rod-like outer segments like other lizards and snakes that inhabit dimly lit environments [3]. A potential explanation for this apparent mismatch between lifestyle and photoreceptor morphology lies in the peculiarities of the microhabitat of *C. nicterus*. Being a sand-burrower means that there are no tunnels or any other open pathways under the surface. Consequently, their visual field is completely blocked, so any photoreceptor would be of little use for anything other than sensing overall light levels. It is thus likely that chemo- and/or mechano-reception are more relevant for the activities that take place under the surface, as has been suggested for the mating behaviour of the closely related species *C. leiolepis* Rodrigues 1991 [50]. In contrast, the openness of the dunes of the middle São Francisco River implies that the light levels are never as low as under forest canopies and can actually be quite high as long as the sky is clear and the moon is present [51]. In this scenario, we hypothesize that the typical diurnal cones might be good enough for brief, occasional excursions above the surface in mildly lit environments.

The examination of freshly extracted retinas showed that, despite the shared morphology of the cones, the oil droplets that they bear in each species are indeed different (Fig. 5), precluding us from making direct comparisons of the number of cone types present in each species based on this feature. The presence and pigmentation of oil droplets is strongly associated with lifestyle in vertebrates; those that live in brightly lit environments, such as many lizards, turtles, fishes, and birds, tend to have several types of oil droplets of different colours [36]. In contrast, the ones that inhabit—or are derived from ancestors that inhabited—dimly lit environments tend to have only transparent oil droplets (e.g., amphibians, geckos) or lack them completely (e.g., teleosts, snakes, mammals) [36, 52]. This correlation has a functional explanation, since colourless oil droplets increase photon catches of cone photoreceptors, while coloured oil droplets decrease it [53]. Thus, the absence of coloured oil droplets probably contributes to maximise the visual sensitivity of the fossorial lizard *C. nicterus* in dim light conditions. On the other hand, the presence of colourless oil droplets compared to other fossorial nocturnal lizards that have lost them, such as pygopodid geckos [39], could be a strategy that allows maximising photon catch, as an alternative to the enlargement of photoreceptor outer segments found in geckos. The presence of oil droplets of different colours in *A. ocellifera* suggests that the cone photoreceptors to which each of them belong have different spectral sensitivities, as observed in many other diurnal lizards [19, 25, 26, 32]. Furthermore, coloured oil droplets enhance the discriminability of coloured objects by narrowing photoreceptor spectral sensitivity [54], suggesting that *A. ocellifera* might have well developed colour vision abilities. Coloured oil droplets are not, however, a requisite for different spectral types of cones or for colour vision, and many vertebrates have them even when they possess only transparent oil droplets (e.g., geckos, frogs) [11, 12, 55], or no oil droplets at all (snakes, mammals) [7, 56]. It is thus possible that there are several spectral types among the cone photoreceptors of *C. nicterus*. That is indeed the case for the closely related fossorial gymnophthalmid *Bachia* cf. *flavescens*, in which at least three different cone opsins have been sequenced [6]. We attempted to obtain spectral sensitivities of visual pigments for our study species using microspectrophotometry. However, as noted by Loew and co-workers [19] and Fleishman and co-workers [28], lizard retinas tend to have a very thick pigment epithelium layer surrounding the photoreceptor outer segments, regardless of dark adaptation. The outer segments tend to remain embedded in the pigment epithelium when this is peeled off the retina, precluding measurements. As a result, we were not able to obtain a minimum number of reliable records of photoreceptor sensitivities in either species. While not a substitute for direct spectral assays, opsin sequencing and/or colour discrimination behavioural experiments could provide insight into the presence of multiple cone classes in the eye of *C. nicterus* and their colour vision abilities.

The ocular media of both species are highly transmissive in the UV region of the spectrum, even by lizard standards [33]. In the case of *C. nicterus*, the lower λ_T50_ value compared to that of *A. ocellifera* is most likely due to the difference in the lens diameter, as there is a well-documented correlation between eye size and λ_T50_ [57]. Our characterisation of the ocular media transmittance in *C. nicterus* is incomplete due to the failure to obtain isolated corneas. However, it has been shown that the lens sets the limit for this property in other lizards [33], and pigmented corneas have only been found in fishes so far [58, 59], so we assume that the cornea has no influence on the overall ocular media transmittance. High UV transmittances correlate well with the presence of UV-sensitive visual pigments in many vertebrates [57, 60] and suggest the potential for UV-vision, which has indeed been demonstrated in several diurnal lizards [28, 32, 33]. On the other hand, all vertebrate photoreceptors have a significant sensitivity to UV light -regardless of the position of their spectral sensitivity maxima- [61] and the absorption of short-wavelength photons by the ocular media has a considerable impact on their overall quantum catches. This is particularly relevant for crepuscular animals, because the spectral composition of light at sunset has a relatively high proportion of short wavelengths compared to other times of the day [62]. Furthermore, in dim light conditions the visual system functions close to its sensitivity limit, and the removal of even a small proportion of photons by the ocular media can affect its activity. Thus, the high UV transmittance of the ocular media of *C. nicterus* likely maximises their visual sensitivity in dim light conditions by allowing all available photons to reach the retina.

Our study is the first one to describe the detailed internal anatomy of the eye of gymnophthalmid lizards. The sand-burrowing, limbless anguimorph lizard *Anniella* is described by Walls [36] as having eyes less than one mm in diameter, with “normal retina and visual cells” without further details. This loose description combined with our findings in *C. nicterus* raises the possibility that, among fossorial lizards, those that burrow in loose soil such as sand and have some occasional aboveground activity are likely to retain functional eyes. Further research on other under-studied clades of sand-burrowing limbless lizards, such as the scincoids *Scelotes* from Mozambique and *Lerista* from Australia would shed light on this topic and provide hints about potential convergences in the visual system associated to convergences in lifestyle, locomotion and microhabitat use. Furthermore, such comparative studies would allow starting to map the association between the loss or retention of particular ocular anatomical structures and divergence times of the fossorial species from their surface-dwelling relatives.

## Conclusions

Our work shows that the fossorial lizard *Calyptommatus nicterus* is remarkably similar, in the functional anatomy of its eye, to other lizards that are diurnal and surface dwelling. These results challenge the commonly held assumptions about loss of visual function in fossorial lizards, which are based almost solely on external eye morphology. Furthermore, our data suggest that vision can be relevant for this species during periods of above-surface activity, particularly if light levels are relatively high as can happen at dawn/dusk under clear skies in an open environment. Finally, our results showcase the need of functional anatomy studies on the visual systems of different lineages of fossorial lizards to understand the range of adaptations or regressions that have evolved in scenarios involving different microhabitats and diel activity patterns.

## Methods

### Animals

Adults of *Calyptommatus nicterus* and *Ameivula ocellifera* were captured in Vacaria (10°39′S, 42°36′W), on the right margin of São Francisco River in the state of Bahia, Brazil (see [63] for a map of the region). They were transported to the laboratory in São Paulo in plastic containers with substrate from the capture site, euthanized by intraperitoneal injection of 2% lidocaine and decapitated immediately for the procedures described below. In some cases, photographs were taken using a stereomicroscope at different stages of dissection of the fresh material to document eye anatomy and dimensions. Details on the identity and number of animals used for each of the procedures described below are provided in Table 1.

**Table 1:** List of specimens used during the study

Species	Sex	Used for	Field N°
*C. nicterus*	Female	Histology	MTR37654
*C. nicterus*	Female	Oil droplet MSP	MTR37659
*C. nicterus*	Female	Oil droplet MSP	MTR37660
*C. nicterus*	Female	OMT	MTR37661
*C. nicterus*	Female	Oil droplets in fresh retina	MTR37662
*C. nicterus*	Female	OMT	MTR37664
*C. nicterus*	Female	Histology	MTR37665
*C. nicterus*	Female	Oil droplets in fresh retina	MTR37666
*A. ocellifera*	Female	OMT, oil droplets in fresh retina, histology	MTR37667
*A. ocellifera*	Male	OMT, oil droplet MSP	MTR37668

*OMT* ocular media transmittance measurements, *MSP* microspectrophotometry

### Histology

The whole heads of two *C. nicterus* and one *A. ocellifera* were fixed in 4% paraformaldehyde in phosphate-buffered saline (PBS) 0.01 M for 4 h and rinsed overnight in PBS at 4 °C. Then we decalcified them by immersion in 7% EDTA for 24 h, rinsed in distilled water, dehydrated in an ascending ethanol series, and embedded in glycol-methacrylate resin (Historesin Embedding Kit, Leica, Wetzlar, DE) following the instructions of the manufacturer. Transverse sections of the head region containing the eyes were cut at 2 μm, mounted on clean glass slides, stained for 1 min with Azure II – Methylene Blue on a hot plate [64], mounted with Entellan (Merck, Darmstadt, DE), and photographed under a light microscope.

### Oil droplet morphological characterisation

After decapitation, the eyes were enucleated and excised and the lens removed. The exposed retina was detached from the eyecup, separated in several pieces in the case of *A. ocellifera*, placed on a glass slide, teased apart with thin entomological pins if necessary, and coverslipped with a drop of PBS. Oil droplets in the fresh preparation were identified on the basis of their round shape and localization inside the photoreceptors at 40X magnification. Several representative regions in each retina were photographed to document the presence and proportions of droplets of different sizes and colours.

### Oil droplet spectral analysis

Animals were dark adapted for a minimum of three hours after which they were sacrificed, and their eyes enucleated under dim deep red illumination. Retinas were extracted in PBS with 6% sucrose and teased from the pigment epithelium. A small piece of the retina was cut, placed on a n.1 cover slip in a drop of the same buffer, and fragmented with fine tungsten needles. The preparation was then covered with a second coverslip and sealed with Corning silicone grease. The retina was placed on the stage of the microspectrophotometer and examined with infrared illumination through an IR-sensitive camera. The single beam microspectrophotometer is described in detail in [65, 66].

A 2 μm measuring beam was placed inside the oil droplet and optical density was measured at 1 nm intervals in two passes, from 350 nm to 750 nm and back. Records fell into three discrete spectral classes (colourless, green-yellow, orange oil droplets). Spectra of coloured droplets were converted to transmittances and the corresponding ‘cut-off’ wavelength (λcut) was obtained as the wavelength of the intercept at minimum transmittance by the tangent to the transmittance curve at 50% of its maximum [67]. For each class of coloured oil droplets, we calculated the mean, mode and range of λcut and peak optical density.

### Ocular media transmittance

We measured the light transmittance of both the isolated lens and spectacle and the whole ocular media in one piece, using the approach of Lind and co-workers [68], as follows. We enucleated the eye, removed the cornea with a circular cut along the *ora serrata*, and gently lifted the lens from the eyecup, cutting through the vitreous when necessary. We placed the samples in a custom-made matte black plastic container (12 mm diameter × 10 mm height) with a circular (5 mm), fused silica window in the bottom filled with PBS. Light from an HPX-2000 Xenon lamp (Ocean Optics, Dunedin, FL) illuminated the samples via a 50 μm light guide (Ocean Optics) through the fused silica window and transmitted light was collected by a 1000 μm light guide connected to a Maya2000 spectroradiometer controlled by SpectraSuite v4.1 software (Ocean Optics). The light guides were aligned with the container in a microbench system (LINOS, Munich, DE). The reference measurement was taken from the container filled with PBS. We took three measurements from each sample, averaged them, smoothed the curve using an 11-point running average and normalized to the highest value within the range 300–700 nm. Finally, we averaged the curves from the two eyes for each species. From these data, we determined the wavelength at which the light transmittance was 50% of the maximum (λ_T50_), a commonly used parameter for comparison of light transmittances [68].

## Data Availability

All data generated or analysed during this study are included in this published article.

## Abbreviations

MSP: Microspectrophotometry
OD: Optical density
OMT: Ocular media transmittance
PBS: Phosphate-buffered saline
UV: Ultraviolet
